# Maternal and paternal exercise regulate offspring metabolic health and beta cell phenotype

**DOI:** 10.1136/bmjdrc-2019-000890

**Published:** 2020-02-27

**Authors:** Jia Zheng, Ana Barbara Alves-Wagner, Kristin I Stanford, Noah B Prince, Kawai So, Joram D Mul, Ercument Dirice, Michael F Hirshman, Rohit N Kulkarni, Laurie J Goodyear

**Affiliations:** 1 Section on Integrative Physiology and Metabolism, Joslin Diabetes Center, Boston, Massachusetts, USA; 2 Department of Medicine, Brigham and Women’s Hospital, Harvard Medical School, Boston, Massachusetts, USA; 3 Section on Islet Cell and Regenerative Biology, Joslin Diabetes Center, Boston, Massachusetts, USA; 4 Harvard Stem Cell Institute, Harvard Medical School, Boston, Massachusetts, USA

**Keywords:** exercise, offspring, glucose tolerance, beta cell(s)

## Abstract

**Objective:**

Poor maternal and paternal environments increase the risk for obesity and diabetes in offspring, whereas maternal and paternal exercise in mice can improve offspring metabolic health. We determined the effects of combined maternal and paternal exercise on offspring health and the effects of parental exercise on offspring pancreas phenotype, a major tissue regulating glucose homeostasis.

**Research design and methods:**

Breeders were high fat fed and housed±running wheels before breeding (males) and before and during gestation (females). Offspring groups were: both parents sedentary (Sed); maternal exercise only (Mat Ex); paternal exercise only (Pat Ex); and maternal+paternal exercise (Mat+Pat Ex). Offspring were sedentary, chow fed, and studied at weaning, 12, 20 and 52 weeks.

**Results:**

While there was no effect of parental exercise on glucose tolerance at younger ages, at 52 weeks, offspring of Mat Ex, Pat Ex and Mat+Pat Ex displayed lower glycemia and improved glucose tolerance. The greatest effects were in offspring from parents that both exercised (Mat+Pat Ex). Offspring from Mat Ex, Pat Ex, and Mat+Pat Ex had decreased beta cell size, whereas islet size and beta cell mass only decreased in Mat+Pat Ex offspring.

**Conclusions:**

Maternal and paternal exercise have additive effects to improve glucose tolerance in offspring as they age, accompanied by changes in the offspring endocrine pancreas. These findings have important implications for the prevention and treatment of type 2 diabetes.

**Significance of this study**

**What is already known about this subject?**

- Maternal exercise in mice improves adult offspring metabolic health.
- Paternal exercise in mice improves adult offspring metabolic health.

**What are the new findings?**

- The combination of maternal and paternal exercise training had the most pronounced effects on offspring glucose tolerance and insulin sensitivity and resulted in adaptation to the endocrine pancreas.

**How might these results change the focus of research or clinical practice?**

- Regular exercise will be recommended for mothers before and during pregnancy and in fathers prior to conception to help prevent the development of obesity and type 2 diabetes in their children as they age.

## Introduction

The prevalence of obesity and type 2 diabetes has increased dramatically throughout the world, and is now considered a pandemic non-communicable disease.^1^ The International Diabetes Federation estimated that in 2017, a total of 425 million people worldwide had diabetes. This number is expected to rise to 629 million by 2045, implying that 1 in every 11 adults will have diabetes. Moreover, one in seven births is expected to be affected by gestational diabetes.^1^ As such, diabetes represents an enormous burden on individual and public health, and the economy.

In recent years, it has become increasingly clear that susceptibility to obesity and type 2 diabetes is strongly influenced by exposure to an adverse intrauterine environment during early development.^2 3^ In addition, human epidemiological studies,^4 5^ as well as rodent studies,^6–9^ have shown that both maternal^4–7 10 11^ and paternal^8 9 12–14^ environmental exposures are critical factors influencing the development of obesity and type 2 diabetes in offspring. This can lead to a vicious cycle of metabolic dysfunction, where rising rates of obesity, pre-diabetes, and diabetes in individuals of reproductive age are propagated through subsequent generations.

It has long been recognized that exercise has important health benefits for people with type 2 diabetes, and regular physical exercise can delay or prevent the onset of this disease.^15^ Exercise during pregnancy protects against the development of hypertension and excessive weight gain in the mother and lowers the risk of both macrosomia and low birth weight in offspring.^16^ Studies in humans also suggest that physical exercise during pregnancy can improve the health of offspring in infancy and childhood.^16 17^ While these important studies strongly suggest that exercise during pregnancy is important for the health of adolescent offspring, it has not been determined if maternal exercise can reduce rates of diabetes or obesity in adulthood or middle age, stages of life marked by a high risk for the development of metabolic disease.

Studies using rodent models have been important in delineating the effects of parental exercise on the metabolic health of offspring. In our studies of mice, we have observed that maternal exercise can abolish the development of glucose intolerance and reduce both insulin concentrations and body fat in male and female offspring as they age, even if the offspring’s mother had consumed a high-fat diet (HFD) during pregnancy.^18 19^ Moreover, maternal exercise mitigated the impairment of liver function in adult offspring that accompanied maternal HFD feeding.^19^ Other groups have also provided compelling evidence in rodents that exercise training of dams improves the metabolic phenotype of the offspring.^3 20–25^

Studies examining the effects of paternal exercise on offspring health have been limited. One report suggested that longer term voluntary wheel running for 12 weeks, where only male mice that ran ~7 km/24 hours were used in the study, resulted in increased risk for obesity in offspring.^26^ In contrast, another study demonstrated that obese males who underwent a swim exercise training regimen three times/week for 8 weeks fathered female offspring with improved glucose tolerance.^27^ We recently reported that voluntary wheel running exercise in males prior to breeding improves the metabolic phenotype of adult offspring.^28^

Thus, multiple studies indicate that both maternal and paternal exercise have beneficial effects on offspring. Interestingly, little is known about the combined effects of maternal and paternal exercise on the metabolic health of offspring. A major goal of the current study was to determine the effects of combined parental exercise training on offspring health.

Maternal HFD feeding results in the deterioration of pancreatic beta cell function and the development of insulin resistance in adult offspring.^29^ Maternal HFD has also been reported to cause hyperinsulinemia in offspring^18 30^ and increased pancreatic mass and increased islet mass and volume.^30^ In contrast, we found that maternal exercise results in lower insulin concentrations in offspring,^18^ suggesting pancreatic adaptations. Thus, the second major goal of this study was to determine the effects of parental exercise training on pancreas phenotype in offspring. Our data indicate that maternal and paternal exercise in high-fat-fed parents leads to improved glucose tolerance in offspring as they age. This was accompanied by beneficial effects on the endocrine pancreas of the offspring.

## Research design and methods

### Mice and exercise paradigm

For maternal exercise groups and their controls, beginning at 6–7 weeks of age C57BL/6 virgin female mice (Charles River Laboratories) were fed a high fat diet (HFD) (D12492 HFD; 60% kcal from fat; Research Diets). Two weeks prior to breeding, female mice were randomly assigned to singular housing cages with (exercise) or without (sedentary) running wheels. Food intake was measured daily and body weight was measured weekly during this 2-week prebreeding period. For paternal exercise, 7-week-old C57BL6 mice (Charles River Laboratories) were randomly assigned to separate cages with (exercise) or without (sedentary) running wheels for 3 weeks before breeding. These parental exercise paradigms are based on our previous studies showing beneficial effects of maternal and paternal exercise on offspring health.^18 28^ For breeding, one virgin male and two virgin females were housed together for 4 consecutive days in cages without wheels and with free access to HFD. Sedentary male and sedentary female were mated with each other (Sed), or male (Pat Ex) and female (Mat Ex) mice were mated with sedentary controls or with each other (Mat +Pat Ex). After breeding, females were returned to their cages to continue their assigned exercise paradigm. The exercise protocols for dams and sires are shown in online supplementary figures S1A and S2A, respectively. The females consumed HFD throughout gestation and lactation. After parturition, litters were culled to six to seven mice. Thus, four offspring groups were generated: both maternal and paternal sedentary (Sed), maternal exercise only (Mat Ex), paternal exercise only (Pat Ex), and both maternal and paternal exercise (Mat+Pat Ex). Offspring were housed in static cages (sedentary) from birth onwards and fed a chow diet (9F 5020 Lab Diet), and male offspring were studied up to 52 weeks of age. Male offspring were studied because we have previously shown that males have a more pronounced phenotype in response to HFD feeding of dams.^18 19^

10.1136/bmjdrc-2019-000890.supp1 Supplementary data

### Glucose and insulin tolerance test

For intraperitoneal glucose tolerance tests (ipGTT), mice were fasted overnight (12 hours) and baseline samples were taken for glucose and insulin measurements, then the mice were injected intraperitoneally with 2 g D-glucose/kg body weight and blood glucose was measured at 15, 30, 60 and 120 min following injection. Blood glucose was measured in tail vein blood samples using a glucometer (Infinity, US Diagnostics). Insulin was measured by ELISA (Crystal Chem, catalog number 90080). Blood glucose response to the ipGTT was calculated as the area under the curve for each mouse according to the trapezoidal method. For intraperitoneal insulin tolerance tests (ipITT), mice were fasted for 4 hours. Following baseline blood glucose measurement, the mice were injected intraperitoneally with 1 U Humulin R insulin/kg body weight, and blood glucose was measured at 10, 15, 30, 45 and 60 min. Blood glucose response to the ipITT was calculated as the area under the curve for each mouse according to the trapezoidal method.

### Islet morphology and immunohistochemistry

At 3 and 52 weeks of age, one male offspring from each litter was randomly selected. The animals were injected with pentobarbital sodium (90 mg/kg body weight) and blood was collected via heart puncture, then the heart was rapidly removed (n=6 mice/group; representing 6 litters). The pancreas was dissected, weighed, fixed overnight in 4% paraformaldehyde, rinsed in phosphate buffered saline (1x), embedded in paraffin, sectioned and processed for immunohistochemistry. Anti-insulin antibody (catalog number ab7842, Abcam) was used to determine beta cell mass, and anti-glut2 antibody (catalog number GT21-A, Alpha Diagnostic International) was used to determine beta cell size. Calculation of beta cell mass and quantification of beta cell size were performed as described previously.^31^

### Statistical analysis

All data are presented as mean±SEM. When two groups were compared (sedentary dams versus trained dams or sedentary sires versus trained sires), we used unpaired two-tailed Student’s t-test. When four groups were compared we used two-way analysis of variance (ANOVA) followed by Tukey’s post hoc test. This two-way ANOVA was thus used when we compared offspring data. A p value <0.05 was considered statistically significant. Since the parent was treated, the ‘n’ was determined by litter.

## Results

### Exercise paradigm and metabolic profile of dams

Daily running distance was monitored in pregnant females and in those females that bred but did not become pregnant (non-pregnant) during the pregestational (day −14 to day 0) and gestational (G0–G20) periods. Pregnant and non-pregnant controls performed similar levels of voluntary running during pregestation, but as pregnancy progressed, the dams decreased their running distance with the lowest level on the day of delivery (online supplementary figure S1B). However, the dams performed considerable running activity, with 8.5±0.3 km/day of voluntary wheel running during the pregestation period and 3.3±0.4 km/day during gestation (online supplementary figure S1C, *left*). Cumulative distance for the dams was 117.8±6.5 and 65.4±6.8 km for pregestation and gestation, respectively (online supplementary figure S1C, *middle*). Comparison of running activity between non-pregnant and pregnant mice showed that the pregnant dams ran less than the non-pregnant mice (online supplementary figure S1C, *right*). Food intake was measured only during the 2 weeks before breeding and not during pregnancy in order to minimize stress to the dams. At this time point, body weights were significantly lower in the exercise-trained dams compared with the sedentary dams. There was no difference in food consumption during the 2 weeks prior to breeding (online supplementary figure S1D). Because we did not want to excessively stress the dams during pregnancy, ipGTTs were not performed during gestation. Instead, fasting blood glucose and insulin concentrations were measured during the second week of pregnancy. Both fasting glucose and fasting insulin were significantly lower in the exercised dams (online supplementary figure S1E), suggesting that the voluntary wheel running improved the metabolic health of dams. Exercise training in dams had no effect on litter size (online supplementary figure S1F).

### Exercise paradigm and metabolic profile of sires

The sires ran 4.4±0.1 km/day for a cumulative distance of 92.1±15.7 km in 3 weeks (online supplementary figure S2B). These results are similar to a previous study, which analyzed the running behavior of male and female Sprague-Dawley rats and found that females ran significantly more than males.^32^ Exercise training in males resulted in significantly lower body weights (online supplementary figure S2C), while there was no difference in food intake (online supplementary figure S2D). After breeding, the exercised sires had significantly lower fasting blood glucose and insulin concentrations (online supplemetnary figure S2E, F), as well as improved glucose tolerance and increased insulin sensitivity (online supplementary figure S2G, H). These data demonstrate that voluntary wheel running improved the metabolic health of sires.

### Parental exercise improved the metabolic health of male offspring at 52 weeks of age

**Figure 1:**
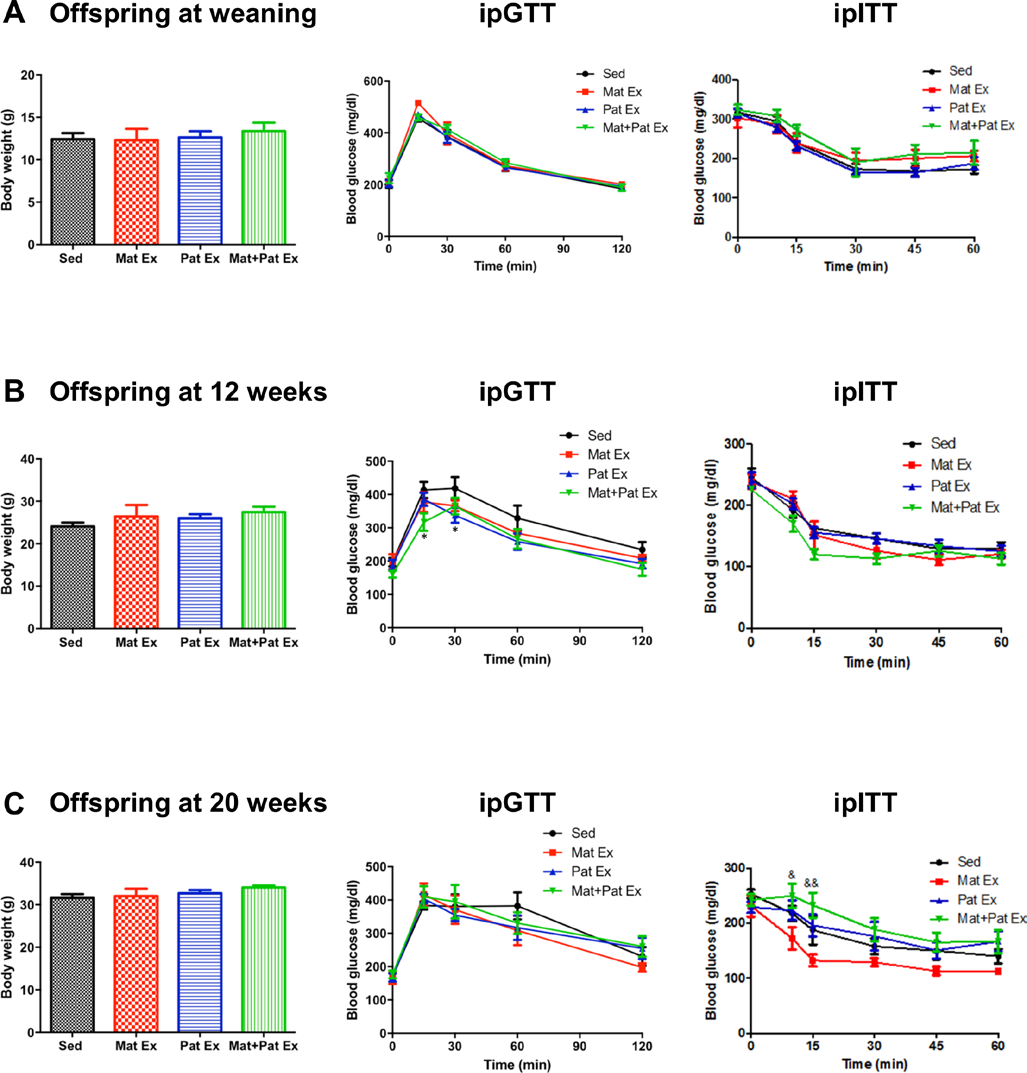
Effect of parental exercise on glucose metabolism in male offspring at weaning (3 weeks), 12 weeks and 20 weeks of age. (A) Body weight (*left*), intraperitoneal glucose tolerance test (ipGTT) (*middle*) and insulin tolerance test (ITT) (*right*) of male offspring at weaning (3 weeks of age). (B) Body weight (*left*), ipGTT (*middle*) and ITT (*right*) of male offspring at 12 weeks of age. (C) Body weight (*left*), ipGTT (*middle*) and ITT (*right*) of male offspring at 20 weeks of age. Data represent mean±SEM. *P<0.05 versus Sed. n=6 litters in each group. AUC, area under the curve; ipITT, intraperitoneal insulin tolerance test; Mat Ex, maternal exercise; Mat+Pat Ex, both maternal and paternal exercise; Pat Ex, paternal exercise; Sed, sedentary.

**Figure 2:**
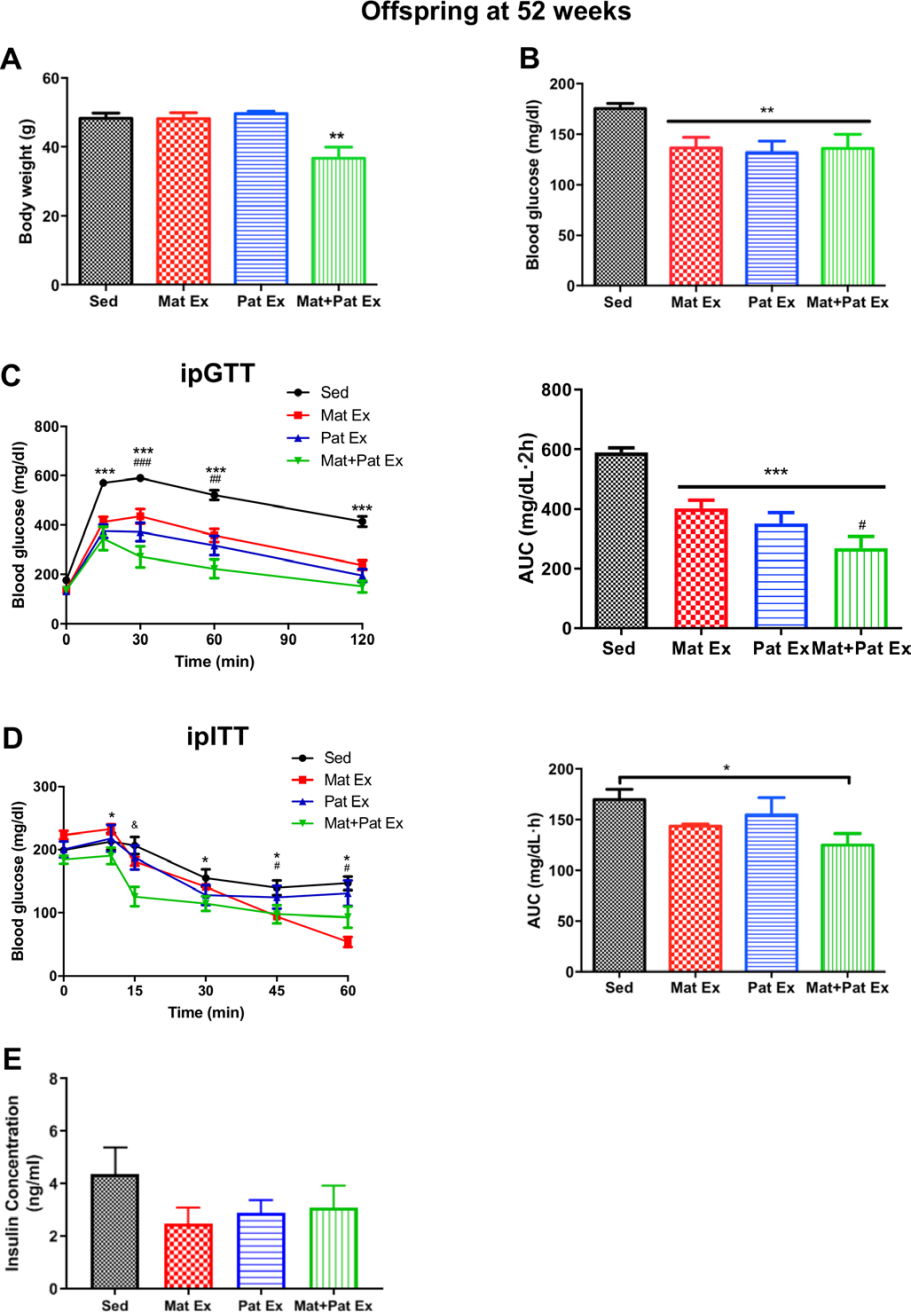
Improved glucose metabolism in male offspring from high-fat fed, exercised parents at 52 weeks of age. (A) Body weight of male offspring at 52 weeks of age. **P<0.01 versus all groups. (B) Blood glucose of male offspring at 52 weeks of age, 6 hours fasted. **P<0.01 versus Sed. (C) Intraperitoneal glucose tolerance test (ipGTT) (*left*) and AUC (*right*) of male offspring at 52 weeks of age. ***P<0.001 Sed versus all groups at 10, 30, 60 and 120 min; ##p<0.01; ###p<0.001. Mat+Pat Ex versus Mat Ex at 30 and 60 min. ***P<0.001 versus Sed; #p<0.05 versus Mat Ex. (D) Intraperitoneal insulin tolerance test (ipITT) (*left*) and AUC (*right*) of male offspring at 52 weeks of age. *P<0.05 Mat+Pat Ex versus Sed at 10, 30, 45 and 60 min; #p<0.05 Mat Ex versus Sed at 45 and 60 min; &p<0.001 Mat+Pat Ex versus all groups at 15 min. *P<0.05 versus Sed. (E) Fasted insulin concentrations of male offspring at 52 weeks of age, 12 hours fasted. Data represent mean±SEM. n=6 litters in each group. AUC, area under the curve; Mat Ex, maternal exercise; Mat+Pat Ex, both maternal and paternal exercise; Pat Ex, paternal exercise; Sed, sedentary.

There is a great interest in comparing maternal, paternal and maternal+paternal exercise training effects on offspring, in the same experiment, in order to determine which one is more important for offspring health. For that, we next investigated the effects of parental exercise on the metabolic health of the offspring. For these studies, we focused on male offspring, since we have previously shown that males have a more pronounced phenotype in response to HFD feeding of dams.^18 19^ There was no effect of parental exercise on offspring body weight, glucose tolerance or insulin sensitivity at weaning (figure 1A), 12 (figure 1B) or 20 (figure 1C) weeks of age. In contrast, at 52 weeks of age, there were multiple effects of parental exercise on offspring. Body weights were significantly lower in offspring from Mat+Pat Ex compared with the three other offspring groups (figure 2A). However, there were no differences in food consumption among all groups of adult offspring. Fasting blood glucose was significantly lower in offspring from all parent groups that exercised (Mat Ex, Pat Ex, Mat+Pat Ex) compared with offspring of parents that were both sedentary (Sed) (all p<0.01, figure 2B). Mat Ex (31.7% lower), Pat Ex (40.5% lower), and Mat+Pat Ex (54.5% lower) all showed improved glucose tolerance versus Sed (p<0.0001, figure 2C), where offspring from Mat+Pat Ex were the most glucose tolerant, with a significantly lower area under the curve compared with the offspring of Mat Ex and Pat Ex, respectively (figure 2C). In this cohort of mice insulin concentrations were not significantly different among the groups (figure 2E), however insulin sensitivity was significantly improved in offspring from Mat+Pat Ex (p<0.05, figure 2D). These results show that if parents are fed an HFD and perform exercise training, there is a significant improvement in glucose tolerance in offspring at 52 weeks of age. Interestingly, the combination of maternal+paternal exercise training had the greatest effect on glucose tolerance in the offspring.

### Combined maternal and paternal exercise alters offspring islet morphology

**Figure 3:**
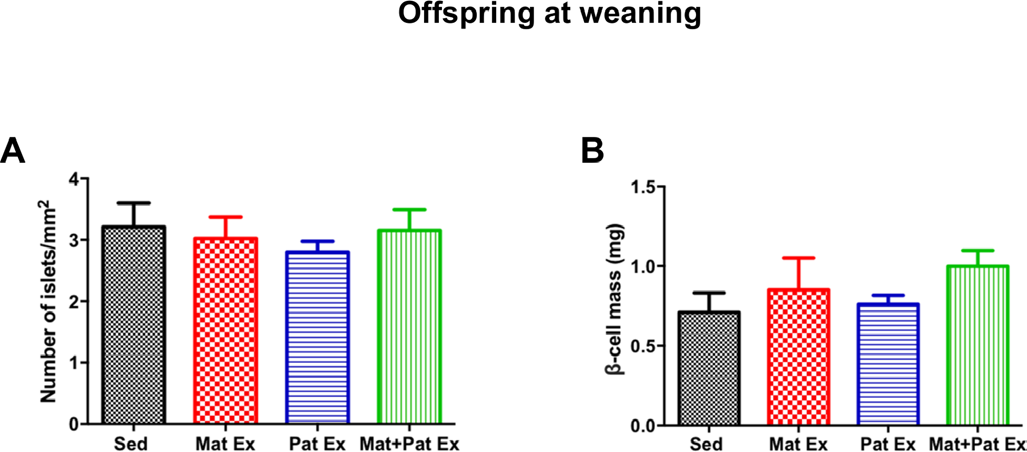
Effect of parental exercise on islet number and beta cell mass in male offspring at weaning. (A) Number of islets/pancreas area (mm^2^). (B) Morphometric analysis of beta cell mass quantification. Data represent mean±SEM, n=5–8 litters in each group. Mat Ex, maternal exercise; Mat+Pat Ex, both maternal and paternal exercise; Pat Ex, paternal exercise; Sed, sedentary.

**Figure 4:**
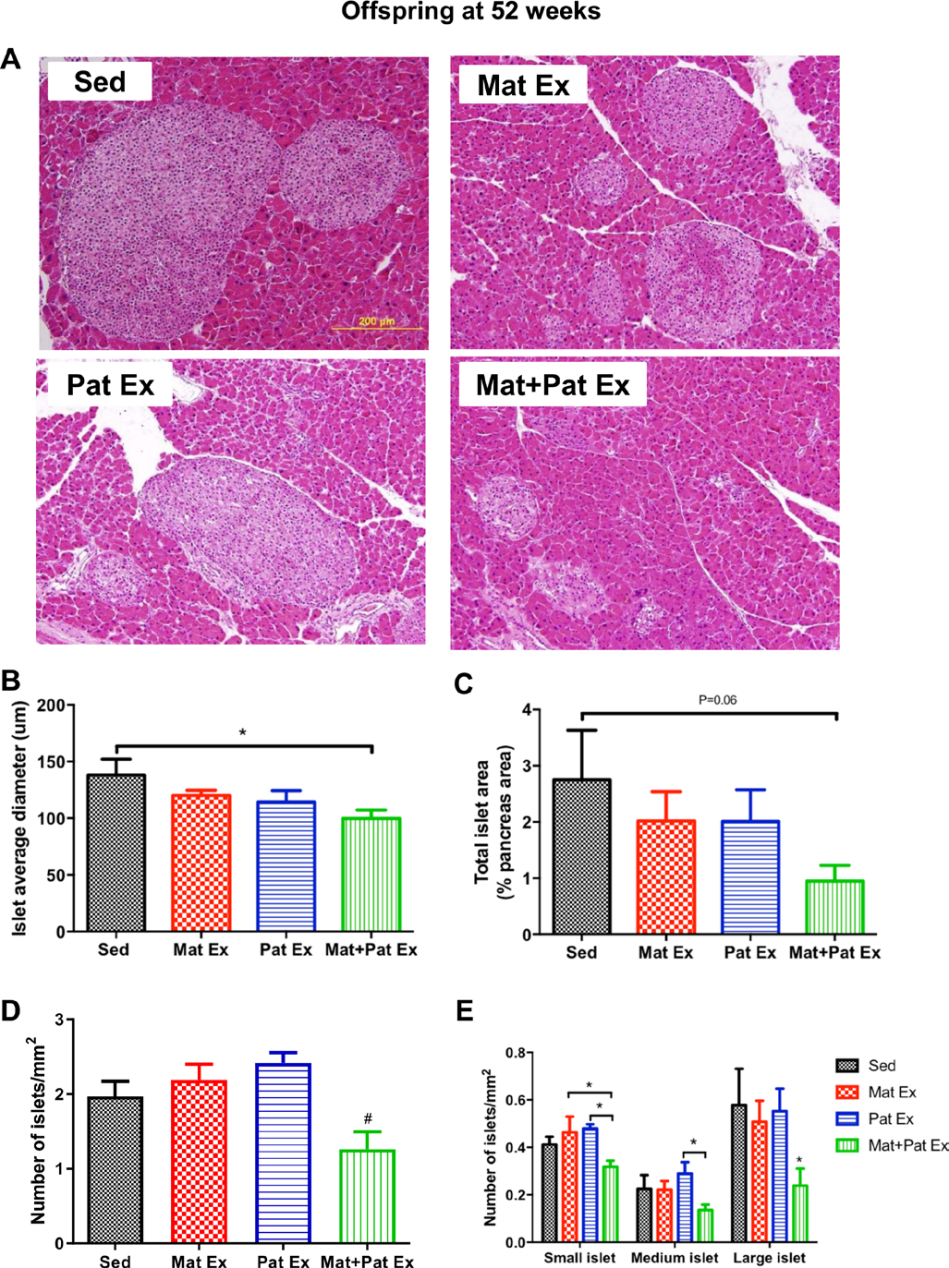
Effect of parental exercise on islet size and density in male offspring at 52 weeks of age. (A) Representative images of H&E staining in sections of pancreas. Scale: 200 µm (20× magnification). (B) Quantitative analysis of islet size was calculated by the average of the longest and shortest diameters of each islet. (C) Morphometric analysis of total islet area expressed as % of pancreas area. (D) Number of islets/pancreas area (mm^2^). (E) Number of different islet sizes calculated as a fraction of the pancreas area (mm^2^). Small islet: 0–5000 μm^2^, medium islet: 5001–10 000 μm^2^, large islet: >10 000 μm^2^. Data represent mean±SEM. *P<0.05 versus Sed, #p<0.05 versus Mat Ex and Pat Ex, n=6 litters in each group. Mat Ex, maternal exercise; Mat+Pat Ex, both maternal and paternal exercise; Pat Ex, paternal exercise; Sed, sedentary.

Since maternal and paternal exercise training showed beneficial effects on the offspring metabolic phenotypes, we determined the pancreatic phenotype of the offspring. There was no difference in the number of islets and beta cell mass in offspring at 3 weeks of age (figure 3). However, at 52 weeks, when there were clear improvements in glucose tolerance, there were changes in the islets in the pancreas of the offspring (figure 4A). For example, islet diameter was significantly decreased (28% lower, p<0.05) in offspring of parents that were both exercise trained (Mat+Pat Ex, figure 4B) compared with the Sed group, and total islet area tended to be smaller in the Mat+Pat Ex offspring (p<0.06 versus Sed, figure 4C). Analyses of the number of islets by pancreas area revealed that offspring from Mat+Pat Ex exhibited lower islet density compared with the three other groups (figure 4D). We further explored the potential differences in the numbers of variable-sized islets in the offspring. Thus, islets were divided based on islet area as follows: small: 0–5000 μm^2^; medium: 5001–10 000 μm^2^; or large: >10 000 μm^2^.^33^ The data indicated fewer numbers of small, medium and large islets in male offspring from HFD-fed, exercise-trained parents at 52 weeks of age (figure 4E). There was no effect of maternal (Mat Ex) or paternal (Pat Ex) exercise training alone on islet diameter or total islet numbers.

### Offspring beta cell phenotype is affected by parental exercise

**Figure 5:**
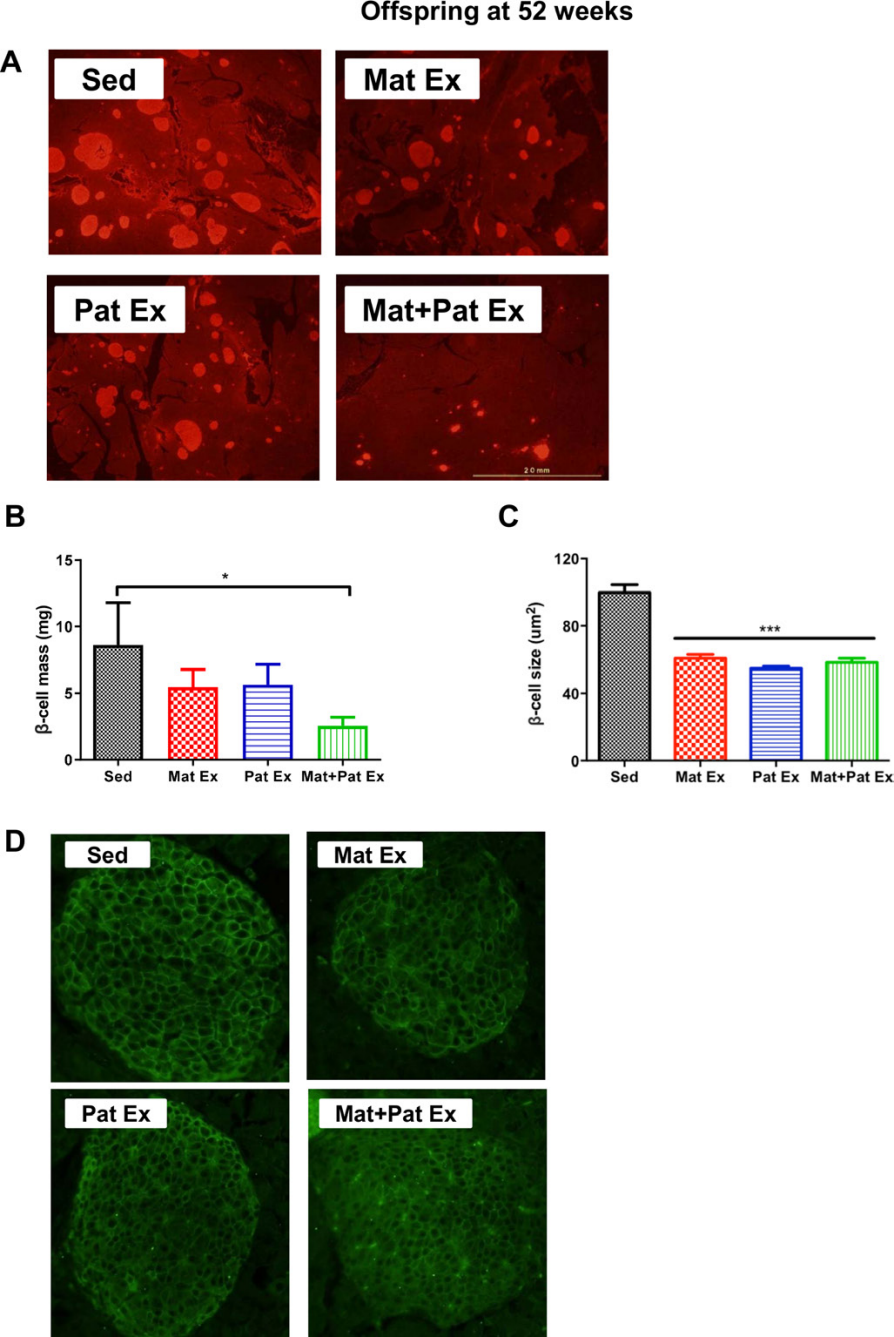
Effect of parental exercise on beta cell mass in male offspring at 52 weeks of age. (A) Representative images of insulin-positive areas in sections of pancreas immunostained with anti-insulin antibody (20× magnification). (B) Beta cell mass was calculated by total beta cell area/pancreas area×pancreas weight (mg). (C) Quantification of beta cell size: 10 islets were randomly selected per mouse and 50 cells were measured (μm^2^) for a total of 500 insulin(+) cells per mouse. (D) Representative immunofluorescence images of pancreas sections stained with anti-glut2 antibody. Images were acquired from all visible multicellular islets at 20× magnification. Data represent mean±SEM. *P<0.05; ***p<0.001 versus Sed. n=6 litters in each group. Mat Ex, maternal exercise; Mat+Pat Ex, both maternal and paternal exercise; Pat Ex, paternal exercise; Sed, sedentary.

To determine beta cell mass, we measured pancreas weights and used pancreas sections for immunostaining for insulin as described previously.^34^ As shown in figure 5A, there were fewer insulin-positive areas in 52-week-old offspring of Mat+Pat Ex, whereas offspring from both Mat Ex and Pat Ex tended to have few insulin-positive areas, compared with the Sed group (figure 5A). When we calculated the beta cell mass by multiplying the relative insulin (+) area (percentage of the insulin-positive area over the total pancreas area) by the wet pancreas weight, we confirmed that combined (maternal+paternal) exercise training resulted in significantly decreased beta cell mass in the offspring (figure 5B, p<0.05 versus Sed). Beta cell size was analyzed by immunostaining the pancreas slides with anti-glut2 antibody in the 52-week-old offspring. Offspring from Mat Ex, Pat Ex, and Mat+Pat Ex all showed a decrease in beta cell size (40% lower, p<0.001) compared with the Sed group (figure 5C, D). Thus, the combination of maternal and paternal exercise training had the greatest effect on beta cell phenotype, with offspring having reduced islet size, islet density and beta cell mass. These results suggest that when combined, maternal and paternal exercise improves offspring insulin sensitivity to maintain glucose homeostasis, resulting in a smaller endocrine pancreas.

## Conclusions

Transmission of obesity and type 2 diabetes phenotypes to subsequent generations is a troubling concept in this age of staggering increases in both the prevalence and costs of these diseases. Fortunately, emerging scientific studies suggest that exercise in parents may be a means to lessen this burden.^3 18–21 23–25 28 35^ Data from murine models show that both maternal and paternal exercise, individually, can have beneficial effects on glucose tolerance and insulin sensitivity in adult offspring.^18 19 28^ Data from human studies show that obese mothers treated by exercise and diet interventions resulted in reduced maternal gestational weight gain during pregnancy and had the potential to decrease adiposity in their offspring at 6 months of age.^36^ These results provide evidence that lifestyle intervention during pregnancy can be a powerful tool to reduce the transmission of increased risk of obesity and diabetes to offspring.

One important question is whether maternal, paternal or the combination of maternal and paternal exercise can result in different adaptations or even in additive effects to improve offspring metabolic health. In this study, we discovered that the magnitude of improvement in mouse offspring metabolic health is similar in response to both maternal and paternal individual exercise. Interestingly, in the current study, we demonstrate that when both parents exercise, there can be an even greater effect of exercise on the metabolic phenotype of the offspring. The combination of maternal and paternal exercise training had a more pronounced effect to improve glucose tolerance in offspring and was the only treatment that improved insulin sensitivity and lowered body weights in the adult offspring. Since weight loss can improve insulin sensitivity,^37^ it is possible that this is an important mechanism for the improved glucose tolerance in the offspring from combined maternal and paternal exercise. Interestingly, we have previously found that the effects of maternal exercise to improve glucose tolerance occurred prior to the effects on offspring body weight. In addition, we found that paternal exercise improved offspring glucose tolerance independent of changes in adult offspring body composition.^18 28^ Thus, it is likely that changes in glucose tolerance with parental exercise are not simply due to changes in offspring body weight or body composition.

In addition to improved glucose tolerance and insulin sensitivity, we found that the combination of maternal and paternal exercise had significant effects on islet morphology and beta cell mass in the offspring at 52 weeks of age. Pancreatic beta cells can generally be described as performing two functions: continuous sensing of blood glucose concentrations and secreting insulin when appropriate.^38^ Regulation of beta cell mass is dynamic and tightly matched to meet the body’s demands for insulin.^34^ Our study found that beta cell mass and beta cell size were significantly decreased in offspring from exercised parents, compared with offspring from the Sed group. One interpretation of these data is that the higher blood glucose concentration in the offspring from the sedentary group promotes compensatory effects that manifest as an increase in beta cell replication and cell size.^39^

In order to understand the mechanisms for the improved metabolic health of offspring from exercised parents, it is important to determine the age at which the glucose metabolism and beta cell function are enhanced. A study in rats reported that maternal exercise improved insulin sensitivity in offspring when they are 10 months old, with no differences in younger rats.^40^ Previous results from our group showed that in offspring from chow-fed dams, maternal exercise did not affect offspring glucose tolerance until 52 weeks of age. However, if offspring developed glucose intolerance as a result of maternal HFD, then prior maternal exercise normalized these effects as early as 36 weeks of age.^18 19^ With paternal exercise, independent of their diet (chow or HFD), male offspring showed improved glucose tolerance at 16 weeks, while female offspring showed an improvement only at 36 weeks of age, with no difference at younger ages.^28^ In the current study, where parents were all HFD fed, we found no differences in the metabolic health and/or beta cell mass of offspring at weaning, consistent with the observations that these phenotypes appear as the offspring age. Thus, both maternal and paternal exercise can modulate the metabolic health of their offspring, with a more robust improvement on glucose metabolism manifesting when the offspring get older, during a period in life when there is an increase in the risk for metabolic disease.

An important ongoing goal is to determine the mechanism(s) that contribute to the improvement in glucose tolerance and insulin sensitivity in the offspring of parents that exercise. Our previous work has determined that decreases in the body weights of offspring are not the direct cause of improved glucose tolerance because changes in glucose tolerance occur prior to changes in body weight.^18^ Epigenetic changes including DNA methylation, histone modification and changes in non-coding RNAs are likely playing a role in regulating the pathways in diverse metabolic tissues in the offspring. Consistent with this concept, in a previous study of paternal exercise we found that exercise training regulates small RNA content of sperm, suggesting that these changes could at least in part be a mechanism responsible for the beneficial effects of paternal exercise on offspring health.^28^ Several tissues have been reported to manifest improved metabolic phenotypes in the offspring in response to parental exercise. For example, we find no effect of maternal exercise on skeletal muscle glucose uptake in male offspring,^18^ whereas pronounced changes are evident in liver function as evidenced by altered gene expression and lower hepatocyte glucose production.^19^ In contrast, we observed an increase in skeletal muscle glucose uptake in offspring from exercise-trained fathers.^28^ Taken together with the current study where we observe changes in the endocrine pancreas of the offspring, we speculate that maternal and paternal exercise are powerful stimuli that affect multiple tissues in the offspring, leading to improvements in glucose homeostasis.

In summary, combined parental exercise promotes improved glucose metabolism in their offspring with significant effects on beta cell mass and size. A better understanding of beta cell physiology will be important in evaluating the significance of intergenerational effects of parental exercise on offspring metabolic health. These findings, if translatable to humans, will have critical implications for the early prevention of obesity and type 2 diabetes.
