# Supplementary material for: Maternal and paternal exercise regulate offspring metabolic health and beta cell phenotype
Source: BMJ Open Diabetes Res Care. 2020 Feb 27;8(1):e000890. doi: 10.1136/bmjdrc-2019-000890 (PMC7050345; doi:10.1136/bmjdrc-2019-000890)

Supplementary Data

Figure S1 - Maternal exercise paradigm and metabolic profile. (A) Time-line of maternal exercise. **(B)** Daily running distance for non-pregnant and pregnant mice (pre-gestational period: Day -14 – Day 0 and gestational period: G0 – G20) with voluntary access to a running wheel. **(C)** The average daily running distance of dams during pre-gestation and gestation period (*left*), the cumulative running distance of mice during pre-gestation and gestation period (*middle*), and the cumulative distance for non-pregnant and for pregnant mice (*right*). **(D)** Body weight change (*left*) and food intake (*right*) of dams before breeding. **(E)** Fasted blood glucose (*left*) and insulin concentration (*right*) of exercised and sedentary dams during the second week of pregnancy, 4-hr fasted. **(F)** Litter size of exercised and sedentary dams. Sed: sedentary, Ex: maternal exercise, G: gestation, AUC: area under the curve. Arrow: breeding start day (4 days without running). Data represent mean ± SEM. *P < 0.05, ***P < 0.001 vs. Sed, n=18-20/group.

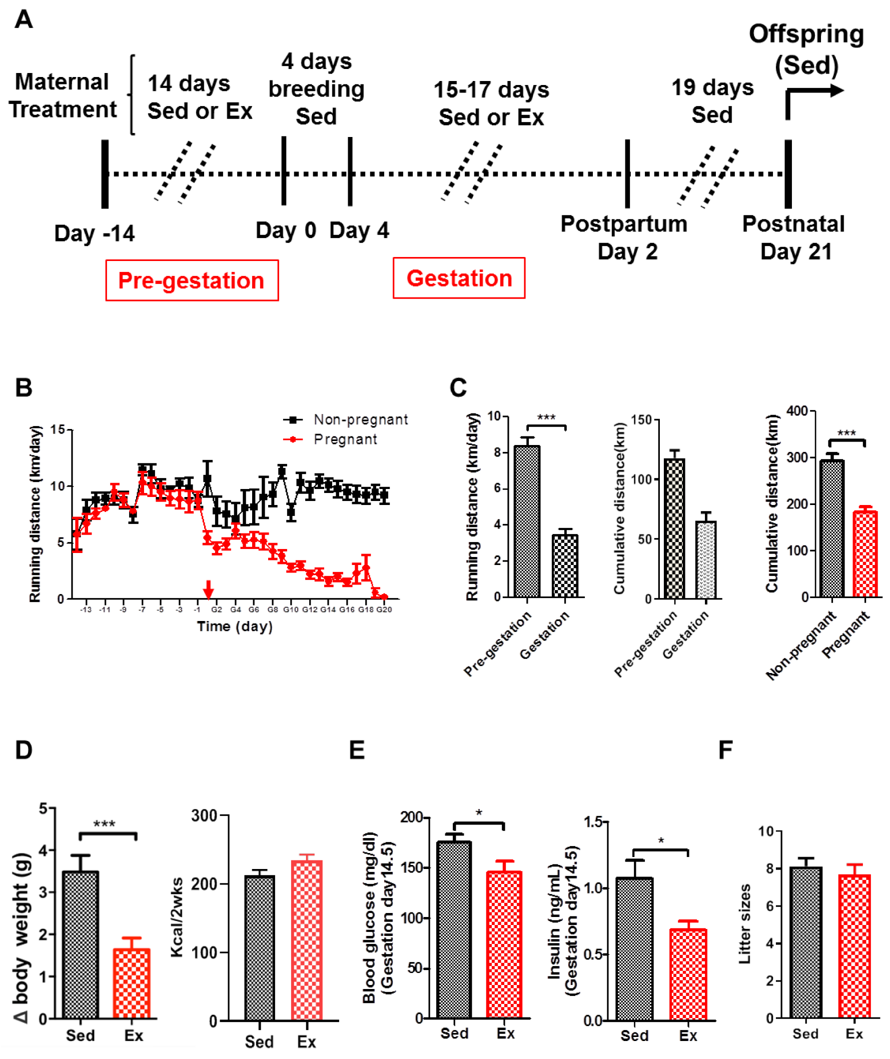

Figure S2 - Paternal exercise paradigm and metabolic profile. **(A)** Time-line of paternal exercise. **(B)** Daily running distance per day (*left*) and cumulative distance during 3 weeks (*right*) before breeding of sires. **(C)** Body weight of sires after 3 weeks of treatment. **(D)** Food intake of sires after 3 weeks of treatment (kcal). Fasted blood glucose **(E)** and insulin concentrations **(F)** of sires after breeding, 6-h fasted. **(G)** Intraperitonial glucose tolerance test (ipGTT) and **(H)** intraperitoneal insulin tolerance test (ipITT) after breeding. Sed: sedentary, Ex: exercise, AUC: area under the curve. Data represent mean ± SEM.*P < 0.05, **P < 0.01, ***P < 0.001 vs. Sed, n=18-20/group.

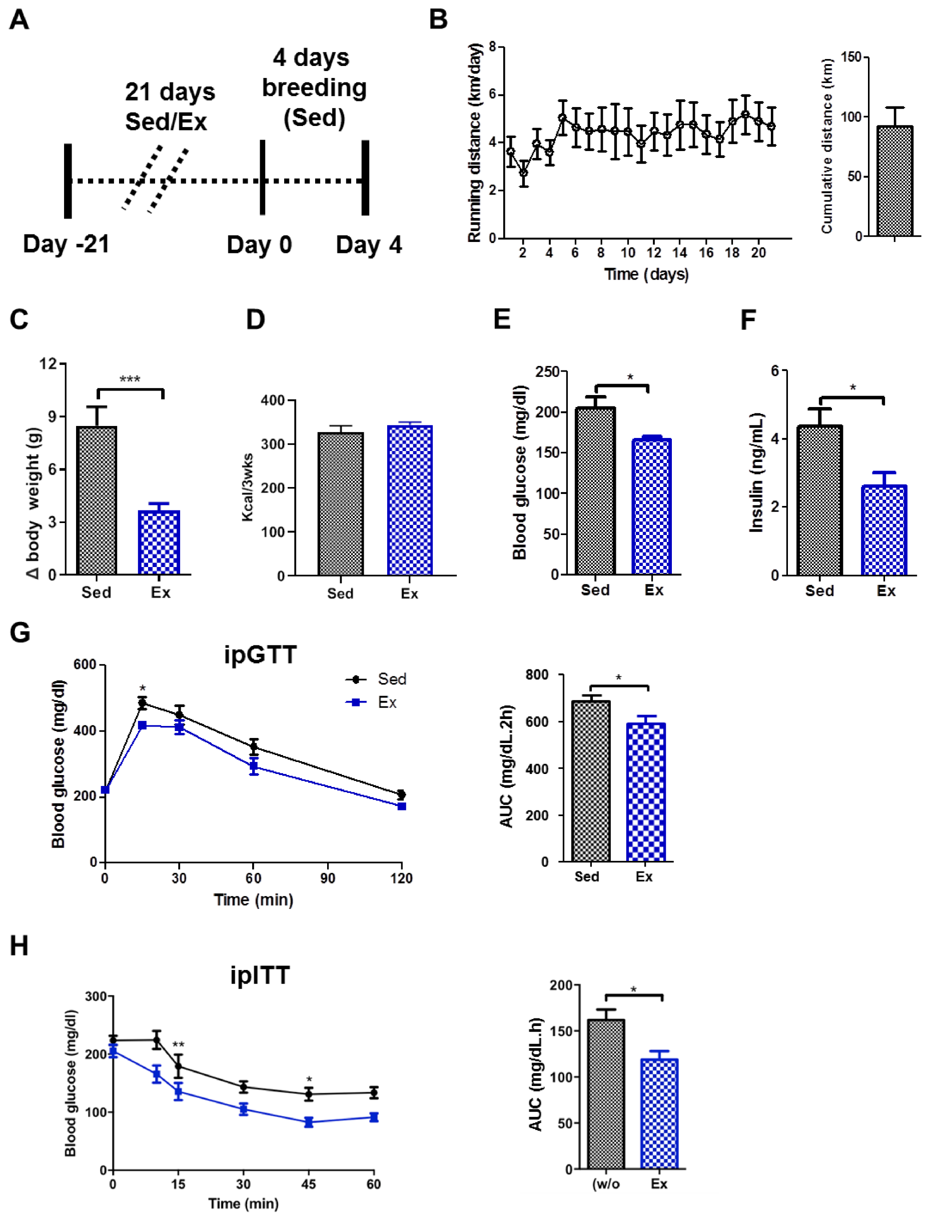

Supplement: Supplementary data [file bmjdrc-2019-000890supp001.pdf]
